# Which reference range should we use for transgender and gender diverse patients?

**DOI:** 10.1210/clinem/dgaa671

**Published:** 2020-09-18

**Authors:** Michael S Irwig

**Affiliations:** Division of Endocrinology, Diabetes and Metabolism, Beth Israel Deaconess Medical Center and Harvard Medical School, Boston, Massachusetts

**Keywords:** clinical laboratory information system, electronic health record, reference range, transgender

Most of us do not think twice when completing a form that requires selecting whether we are male or female. This seemingly benign gender item, however, can cause distress and feelings of exclusion or rejection for our patients who identify as transgender, a broad term that may include nonbinary and gender-queer individuals. Approximately 1 in 200 adults in the United States identify as transgender. The sex/gender item may leave transgender patients wondering whether to select their affirmed gender or the one corresponding to legal documents. What if somebody is nonbinary? Will the selection determine whether the provided services (eg, Papanicolaou test, prostate-specific antigen test) will be covered or denied by somebody’s health insurance?

The binary sex/gender item is usually a mandatory piece of information collected for laboratory information systems. This piece of information determines which reference range should be provided for test results. Although most of our tests do not have different reference ranges based on sex, some do and have the potential to influence diagnosis and management strategies for our patients. In their article entitled “Approach to interpreting common laboratory pathology tests in transgender individuals,” Cheung and colleagues use a case-based approach to illustrate which reference range would be most appropriate for a particular transgender patient in a given scenario (1). The authors propose using the reference range of the affirmed gender once individuals have commenced hormone therapy except for tests that are organ dependent, such as cardiac troponin and prostate-specific antigen.

As Cheung et al describe, the key principles for the selection of the reference range are whether the patient has started gender-affirming hormone therapy and which organs and systems are influenced by hormone therapy. Individuals designated male at birth generally have larger organs (heart, liver, muscle, etc) after male puberty than those designated female, so the reference range differs for tests such as troponin, liver enzymes, and creatinine. Testosterone and estrogen therapy often influence bone density, erythropoiesis, lipid parameters, and chemistry tests such as reproductive hormones, prolactin, and sex-hormone binding globulin.

From a practical standpoint, phlebotomists needs to verify patient identities without misgendering patients. How does the laboratory know which reference range to use for a transgender patient? Rather than defaulting to the sex designated at birth field or to the affirmed gender field in the electronic health record (EHR) (assuming that these fields even exist), Cheung et al propose that ordering clinicians be responsible for providing the gender to be used to the laboratory. The advantage to this approach is that treating providers know the clinical histories of their patients, such as time on hormone therapy and organs present. Disadvantages could be the impracticality of requesting different reference ranges for different tests on the same order (eg, a male troponin reference range and a female hemoglobin reference range) and knowledge gaps among clinicians when it comes to how and which laboratory tests can be altered by hormone therapy. Perhaps an easier approach would be to provide both the male and female reference ranges for transgender patients. More information is better than less and gives clinicians greater flexibility to interpret test results. For example, when using the estimated glomerular filtration rate, which is based on a formula that includes sex, it would be helpful to know whether using one sex instead of the other would influence an important treatment decision such as the dosing of a medication. Another example is using the affirmed gender to calculate *z*-score for bone mineral density (2).

The article by Cheung and colleagues also raises the important question of how the health care system can incorporate gender identity to improve the health care and patient experiences of transgender people. In 2010 the Institute of Medicine recommended that EHRs collect data on gender identity and require this for meaningful-use goals in the United States (3). In 2011 the World Professional Association for Transgender Health convened an EHR working group on this topic (4). Two of its recommendations were for EHRs to add demographic fields for preferred/affirmed name, gender identity, and pronoun and to have a notification system for this information to avoid patient misgendering by clinical staff. To collect gender identity information, the working group recommended a 2-step method that consists of sex assigned at birth and gender identity. This 2-step method performed better than a 1-step method among samples of mainly university students (5). Another recommendation was to maintain an inventory of a patient’s genitourinary organs and whether the patient has breasts. In 2015 the Office of the National Coordinator for Health Information Technology and the Centers for Medicare and Medicaid Services issued rules requiring EHR software certified for meaningful use to include fields for gender identity and sexual orientation (6). Despite this guidance, updating and implementing new EHR fields has proven to be a slow and cumbersome process. At the University of Iowa Hospital and Clinics, it was estimated that more than 100 hours of dedicated time from information technology personnel were needed to implement affirmed names for patients in its EHR (7). This does not count the time and effort of numerous staff and administrators across many departments who participated in meetings and training sessions to carry out this major undertaking.

What are the future directions for using appropriate reference ranges for transgender patients? Although progress has been made in this new area, there is still much work to be done and many systemic barriers to overcome within our practices and hospitals. Starting with the basics, studies are needed to establish reference ranges for transgender populations on hormone therapy. For example, it would be helpful for clinicians to have a reference range for prolactin for patients on estrogen therapy because the modest elevation is rarely clinically significant. From a systems perspective, many EHRs and laboratory information systems need to be updated to capture gender identity because many systems lack this basic capability. Until there is a mandate to do so, the failure to capture gender identity in EHRs will hinder much-needed research on gender-diverse populations, including disparities. Let us not be shy about using our voices and positions to be advocates and allies for our transgender patients to continually improve their care.

## Data Availability

Data sharing is not applicable to this article because no data sets were generated or analyzed during the present study.

## Abbreviation:

EHR: electronic health record.

## Additional Information

***Disclosure Summary:*** M.S.I. has nothing to declare.
